# A Novel Synergistic Association of Variants in PTRH2 and KIF1A Relates to a Syndrome of Hereditary Axonopathy, Outer Hair Cell Dysfunction, Intellectual Disability, Pancreatic Lipomatosis, Diabetes, Cerebellar Atrophy, and Vertebral Artery Hypoplasia

**DOI:** 10.7759/cureus.13174

**Published:** 2021-02-06

**Authors:** S. Charles Bronson, E. Suresh, S. Stephen Abraham Suresh Kumar, C. Mythili, A. Shanmugam

**Affiliations:** 1 Internal Medicine: Diabetes and Endocrinology, Institute of Diabetology, Stanley Medical College & Hospital, Chennai, IND; 2 Department of Neurology, Sree Balaji Medical College & Hospital, Chennai, IND; 3 Biochemistry, Institute of Diabetology, Stanley Medical College & Hospital, Chennai, IND

**Keywords:** ptrh2, kif1a, peripheral neuropathy, axonopathy, pancreatic lipomatosis, outer hair cell, exocrine pancreas, diabetes, cerebellar atrophy, neuro-pancreatic syndromes

## Abstract

The gene PTRH2 encodes a protein with peptidyl-tRNA hydrolase activity and is involved in the translation process in protein synthesis. The kinesin family member 1-A (KIF1A) gene encodes a molecular motor involved in axonal transport along microtubules. Mutations in these genes lead to respective phenotypical conditions that have been reported in the literature. In this paper, we present a novel syndrome of concurrent occurrence of mutations in the PTRH2 and KIF1A genes in a 19-year-old girl of Dravidian-Tamil descent from the Southern part of India. The girl presented with global developmental delay, intellectual disability, weakness of upper and lower limbs, and diabetes. On workup, she was found to have severe peripheral axonopathy, outer hair cell (OHC) dysfunction, severe bilateral sensorineural hearing loss (SNHL), total pancreatic lipomatosis, exocrine pancreatic insufficiency, cerebellar atrophy, vertebral artery hypoplasia, and scoliosis. The patient had a deceased elder sibling who also had had a similar phenotype. Whole exome sequencing (WES) revealed a novel variant in the PTRH2 gene and a rare variant in the KIF1A gene. The predominant axonal involvement seen in our patient, which was attributable to KIF1A involvement, distinguishes this syndrome from the infantile-onset multisystem neurologic, endocrine, and pancreatic disease (IMNEPD) caused by PTRH2 involvement alone. To the best of our knowledge, this is the first report in the medical literature of a syndrome caused by the synergistic occurrence of mutations in the PTRH2 and KIF1A genes. In order to provide more clarity on the genetic and clinical features of such syndromes and to aid the treating clinician to recognize the existence of such syndromes, we propose the broader umbrella term “neuro-pancreatic syndromes” (NPS). Presently, under NPS, we include two entities: the syndrome described by us in this paper and the IMNEPD. Prompt and effective recognition and management of such NPS would immensely benefit the patient in terms of treatment and prognosis. Furthermore, we hope that this paper will promote further understanding of NPS and foster more research, both clinical and genetic, which would widen the spectrum of NPS. Eventually, this would throw more light on treatment options and ultimately benefit patients with NPS.

## Introduction

The gene peptidyl t-RNA hydrolase-2 (PTRH2) on chromosome-17 encodes a highly conserved mitochondrial protein with peptidyl t-RNA hydrolase-2 enzymatic activity, which is involved in the efficient conduction of the translation process [1]. The gene kinesin family member 1-A (KIF1A) codes for a molecular motor belonging to the kinesin superfamily, which is involved in axonal transport along microtubules [2]. Biallelic mutations in PTRH2 were observed to be associated with a phenotype of intellectual disability, post-natal microcephaly, progressive ataxia, muscle weakness, demyelinating neuropathy, exocrine pancreas insufficiency, sensorineural hearing loss (SNHL), and hypothyroidism, and it was termed the infantile-onset multisystem, neurologic, endocrine and pancreatic disease (IMNEPD) [3]. KIF1A-related disorders (KRDs) include a wide phenotype of spasticity, axonal sensorimotor neuropathy, congenital contractures, dystonia, dysautonomia, seizures, neurodevelopmental delay, optic atrophy, corpus callosal dysgenesis, and cerebellar atrophy [4,5].

Diabetes that occurs in children and adolescents usually has wide differentials including type-1 diabetes, type-2 diabetes, and rarer syndromes that include diabetes as a component. Thus, diabetes in the young poses a confounding picture and diagnostic challenge to the treating clinician [6]. In this article, we discuss how we traced an apparent case of type-1 diabetes mellitus (T1DM) to a complex syndromic association that was related to concurrent mutations in both the PTRH2 and KIF1A genes in the same patient. The variant in PTRH2 we report here is a novel one, hitherto unreported, and the variant in KIF1A is a rare one. To the best of our knowledge, this is the first report in the medical literature of such a novel syndrome arising due to co-occurring mutations in both the PTRH2 and KIF1A genes with a synergistic effect on the phenotype.

Furthermore, we propose the broader term “neuro-pancreatic syndromes” (NPS) to cover the syndromes that share similar features, and all the more to raise awareness among the practitioners, especially at the point of first care, about the existence of such syndromic conditions.

## Case presentation

The patient we describe in this paper was a 19-year-old girl of Dravidian-Tamil descent, from Southern India. She was initially referred to us from another hospital when she was 14 years of age, as a case of the then newly detected T1DM. The girl had had a history of difficulty in walking and using her hands even from childhood. She also had a poor scholastic performance and was attending a school for special children. At the time of initial detection of diabetes at the other hospital, the girl had presented with a history of abdominal pain for 10 days and fever and chills for three days. She had been found to have random blood glucose (BG) of 526 mg/dl (29.1 mmol/l), ketosis, and HbA1c of 14.3% (133 mmol/mol). She was treated with rehydration measures and insulin; her condition improved and was discharged. Afterward, she was referred to us with a diagnosis of T1DM. We treated her with subcutaneous basal-bolus insulin therapy. The C-peptide response was inadequate. The anti-glutamic acid decarboxylase-65 (GAD-65) autoantibody was negative. Her insulin requirement at that time was around 1.3-2 units/kg/day. In the following months, a baseline workup was done. Complete blood count and renal and liver function tests were within normal limits. An ultrasonogram of the abdomen showed a fatty liver. As the girl had weakness of upper and lower limbs along with intellectual disability, a neurology consultation was done. Nerve conduction study (NCS) pointed to a ‘severe axonal sensory and motor neuropathy of both upper and lower limbs’. Nerve biopsy revealed a ‘chronic uniform axonopathy consistent with hereditary axonopathy’. Areas of axonal degeneration and demyelination were observed. Muscle biopsy was unremarkable. The girl was thus diagnosed to have a probable hereditary neuropathy and was advised to undergo supportive measures like physiotherapy. Subsequently, she was being treated as a case of T1DM with hereditary neuropathy and was under follow-up.

Over the next few years, we could observe that her insulin requirement was progressively coming down. Often, her post-prandial BG was found to be lower than the fasting BG, sometimes even reaching hypoglycemic levels after a meal. Though, initially, we thought that it could be a honeymoon phase, the persistently lower need for insulin directed us to think otherwise. Eventually, prandial insulin was stopped and she was maintained only on basal insulin. Gradually, the requirement reached about five to six units per day (around 0.17 units/kg/day) of basal insulin alone. At this juncture, we decided to evaluate the case further.

**Figure 1:**
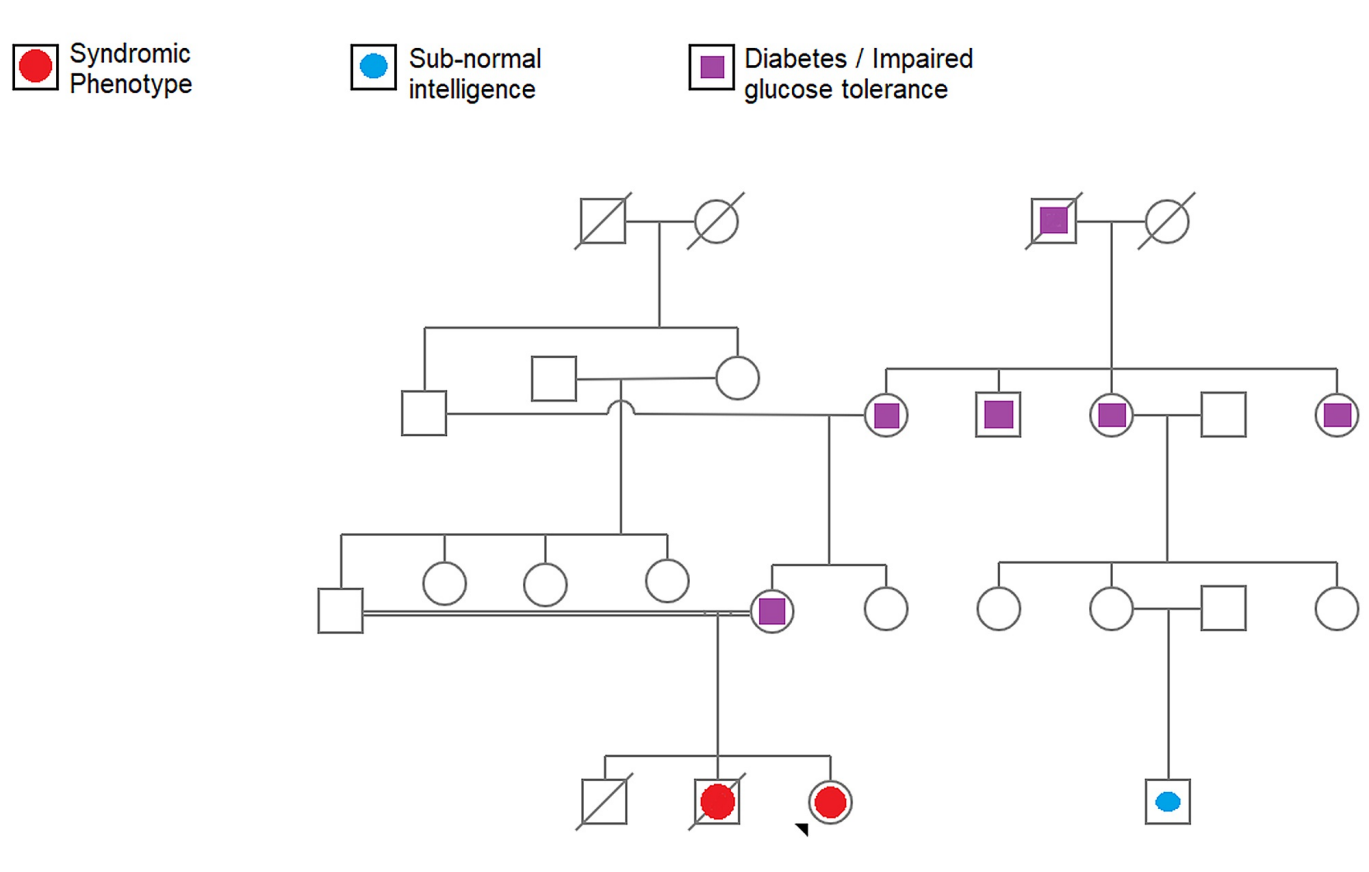
The pedigree chart showing the occurrence of the phenotype in the family

A detailed history of the case was elicited from the girl’s mother. According to that, the girl had been born of a consanguineous marriage. Her father and mother were first cousins. The firstborn child of the couple had been a male who had died one week after birth. The cause of death was not known. The second child had also been a boy who had lived up to two-and-a-half years of age, had experienced recurrent fevers, and had died due to suspected tuberculosis. He had been delivered a few weeks before term by cesarean section due to premature rupture of membranes. The boy had had thin and weak limbs and had a history of developmental delay, notably delayed speech and walking. He had barely started to sit up from lying position just a few months before he passed away. He had been able to say only a few mono- and bi-syllabic words till then. The girl in this report was the third child. She had been delivered at term by cesarean section. There had been no history of any fever in the mother during the antenatal period, difficult labor, or perinatal complications. The girl had had a global delay in attaining developmental milestones including major motor, language, and cognitive milestones. She had started to walk at around one-and-a-half years of age. She had walked with a broad gait, with difficulty in maintaining balance, and experienced frequent falls. As she had grown up, she had faced difficulty in using her hands to hold things and difficulty in gripping her footwear. She had been unaware if her footwear slipped. These weaknesses had seemed to progress till her early teenage years. The girl had attended regular school for a few years where she had been observed to have sub-normal intelligence and poor scholastic performance. Hence, she had been transferred to a school for special children. She had had a history of passing oily and sticky stools even from her infancy and this had been noticed by the mother a few months after the girl's birth. Three days after her birth, the girl had experienced an episode of a bout of bleeding from her mouth and nostrils, for which she had been treated in the neonatal care unit. A similar episode of vomiting of suspected altered colored blood had happened at eight years of age. There had been no further episodes since then. At the age of seven years, the girl had reportedly had an episode of tonic posturing of upper limbs, after which she had been given clobazam for a year and then stopped. She had not had any similar episodes since then. When probed further, the mother said that the girl had seemed to be hard of hearing, which had not been very evident due to her intellectual impairment. Furthermore, the mother also revealed that her deceased second child also had had a history of passing oily stools, recognized a few months after birth, and it had been more severe than that with the girl. The girl had no history of decreased vision, photophobia, or nyctalopia. She had attained menarche at 16 years of age and continued to have regular menstrual cycles. There was a history of diabetes in relatives on the maternal side of the family. There was also a history of subnormal intelligence alone in a second cousin of the girl (Figure 1).

**Figure 2:**
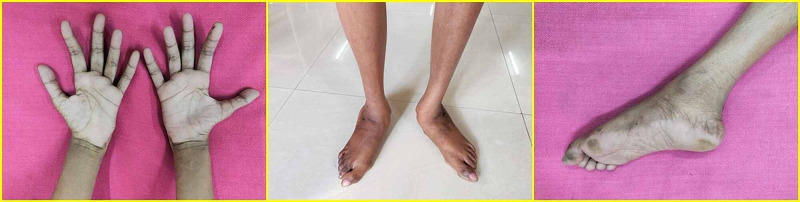
Wasting of muscles in the hands, legs, and feet. Pes valgus and Pes cavus abnormalities in the feet

On examination, the girl was thin, was 152 cm tall, weighed 34 kg, and had a BMI of 14.7 kg/m^2^. Her blood pressure was 110/70 mmHg; pulse was 80/min and regular in rhythm. She had a friendly demeanor and was co-operative. She could hear loud spoken words and was able to answer the questions posed, in a simple yet meaningful way. Her teeth were stained and a few were broken reportedly due to falls during childhood. She walked with a broad, high-stepping, stomping gait that involved both lower limbs. Her upper and lower limbs were thin. There was wasting of muscles in hands, legs, and feet, with mild clawing of fingers, and a pes cavus and valgus deformity of the feet (Figure 2).

The girl also had scoliosis of the vertebral column. There was a progressive symmetrical decrease in muscle power and sensation towards the distal part of both the upper and lower limbs (distal > proximal). Muscle power was 5/5 at the shoulder; 4/5 at the elbow; 3+/5 at the wrist; 3/5 at the small joints of the hand; handgrip was present, yet weak. In the lower limbs, power was 5/5 at the hip; 3+/5 at the knee; 3+/5 for plantar flexion, and 0/5 for dorsiflexion of the foot. Among the deep tendon reflexes, triceps-jerk was diminished, biceps- and supinator-jerks were absent, knee-jerk was diminished, and ankle-jerk was absent. The plantar reflex was absent. Sensory perception including touch, pain, and joint position sense was normal at the proximal part of the upper and lower limbs, with a gradual diminution toward the distal end. However, it must be noted that owing to the intellectual impairment of the patient, it was difficult to conduct and interpret the sensory examination. There was no nystagmus. There was no dysdiadochokinesia or intention tremor. There was a mild degree of past-pointing in finger-nose and finger-nose-finger tests. Tandem walking was not possible. The girl was able to stand keeping her feet together, albeit with difficulty, with the eyes open. When she was asked to close her eyes, Romberg’s sign was positive. Overall, the lower limbs were more severely involved than the upper limbs. The abdomen was soft and non-tender. Clinical examination of the cardiovascular and respiratory systems was normal.

Clinico-psychological assessment of the girl showed an intelligence quotient (IQ) of 43 and a moderate intellectual disability. Fundus examination showed a normal ophthalmic fundus, with no retinopathy or degeneration. The Rinne test showed that air conduction was better than bone conduction. The Weber test was lateralized equally on both sides. Pure tone audiometry was done and it revealed a bilateral, severe, sloping SNHL. While the auditory brainstem response testing showed no retro-cochlear pathology, the otoacoustic emissions testing pointed to an inadequate outer hair cell (OHC) functioning in both ears. High-resolution CT of temporal bones was normal.

**Figure 3:**
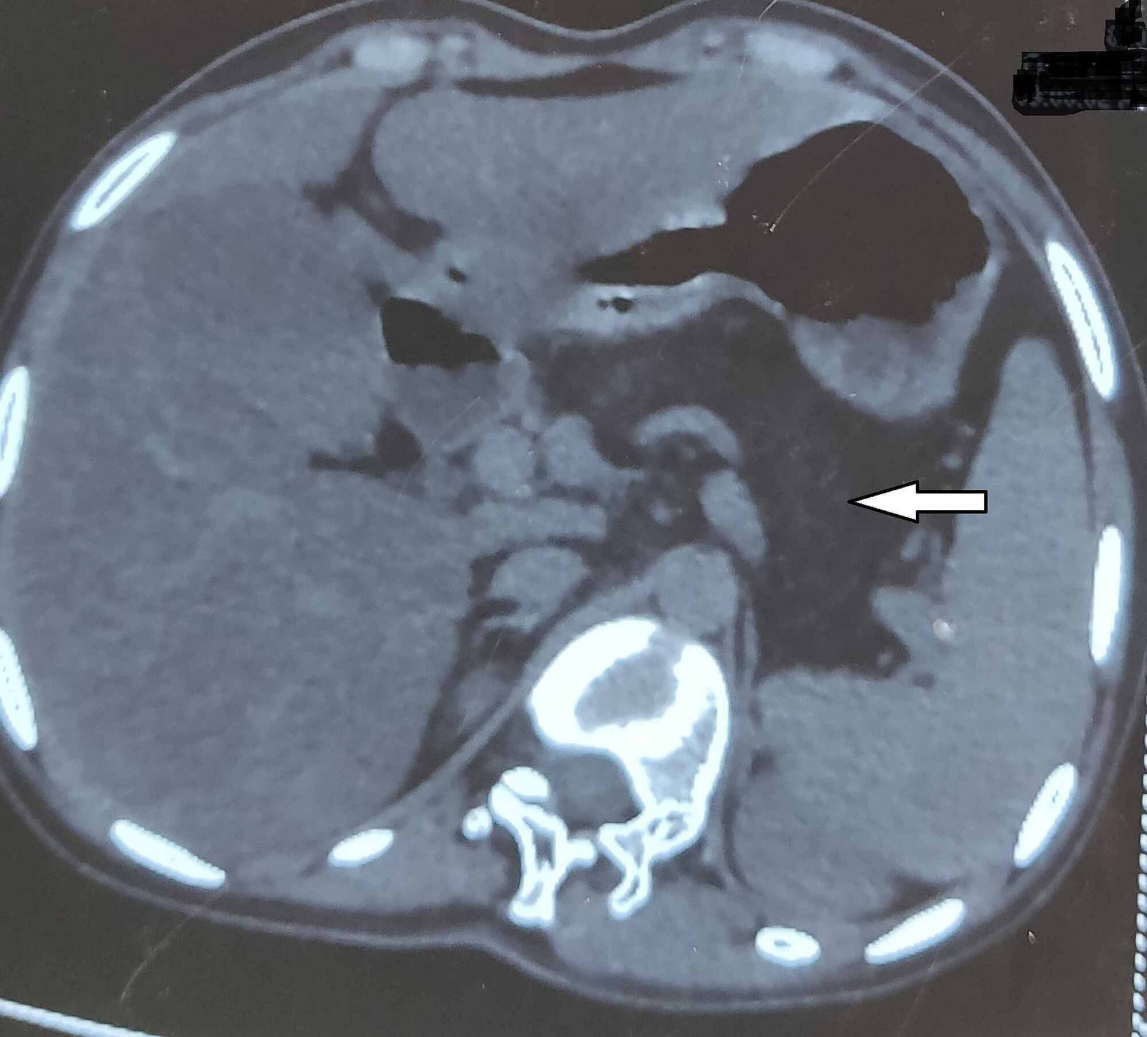
CT of the abdomen showing total pancreatic lipomatosis CT: computed tomography

Contrast-enhanced CT of the abdomen was done and it revealed that the entire pancreas was replaced by fat with preservation of the architecture of the pancreatic duct, the condition termed as ‘pancreatic lipomatosis’ (Figure 3).

There was no calcification or atrophy of the pancreas. Fatty liver was observed. Upper gastrointestinal endoscopic examination showed no abnormality. Chest X-ray, electrocardiogram, and echocardiogram were normal.

Laboratory investigations showed the following results: fasting BG: 118 mg/dL (6.6 mmol/L); post-prandial BG: 163 mg/dL (9.1 mmol/L); HbA1c: 7.7% (61 mmol/mol). Complete blood count was within normal limits, though the mean corpuscular volume (MCV) was at the higher end (97 fL), the reference range being 80-101 fL. Peripheral smear showed a normocytic, normochromic blood picture. Renal and liver function tests and serum electrolytes were within normal limits. Serum calcium (8.7 mg/dL) was near the lower end of normal. Serum phosphorus was normal. Serum creatine kinase and lactate dehydrogenase levels were normal. The C-peptide response (fasting: 1.9 ng/mL; post-meal: 2.3 ng/mL) had improved when compared to the initial years of the detection of diabetes. Fasting lipid-profile showed a ‘hypolipidaemic’ picture with total cholesterol of 86 mg/dL; triglycerides of 56 mg/dL; high-density lipoprotein of 34 mg/dL; low-density lipoprotein of 41 mg/dL; and very-low-density lipoprotein of 11 mg/dL. Serum amylase (4.8 U/L) and lipase (2.1 U/L) were lower than normal levels. Serum lipoprotein electrophoresis did not show any specific abnormality. Serum folic acid, iron, and ferritin levels were within normal limits. Serum vitamin B12 level was low (less than 100 pg/ml, the normal reference range being 197-771 pg/ml). Anti-intrinsic factor and anti-parietal cell autoantibodies were negative. The vitamin D level was 6.49 ng/ml and it was in the deficient range. Prothrombin time was mildly elevated (16.1 seconds) while the international normalized ratio (1.14) was in the upper range of normal. Thyroid function tests showed a mildly elevated thyroid-stimulating hormone (TSH) (5.57 µIU/mL) while free T3 and T4 were within normal limits. Anti-thyroid peroxidase and anti-thyroglobulin autoantibodies were negative. There was no proteinuria. Stool examination showed the presence of fat globules.

**Figure 4:**
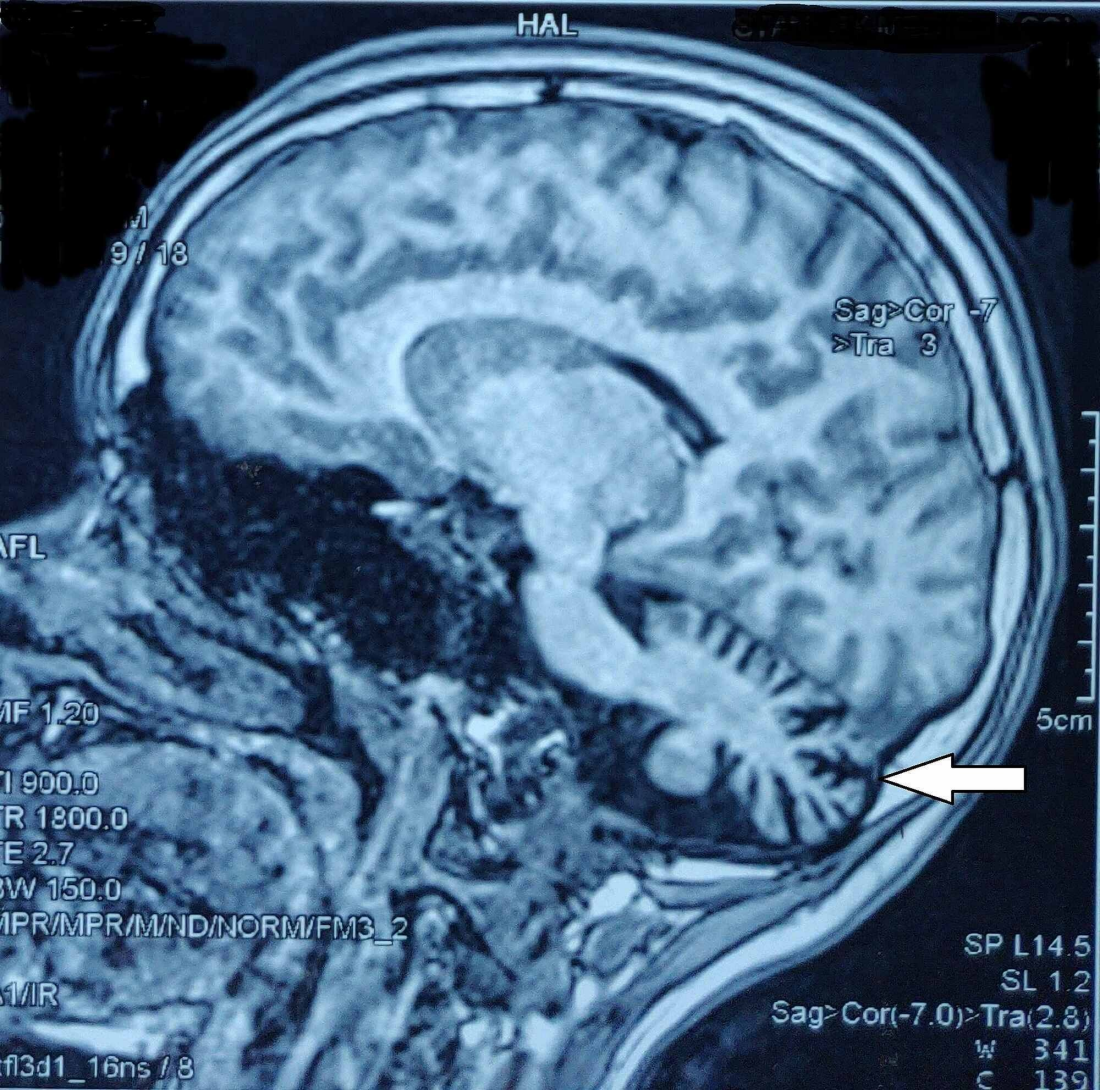
Cerebellar atrophy on MRI of the brain (arrow) MRI: magnetic resonance imaging

MRI of the brain showed volume loss and prominence of folia of the cerebellum suggestive of cerebellar atrophy (Figure 4).

Additionally, magnetic resonance angiography showed the presence of a hypoplastic right vertebral artery. The spinal cord was normal on MRI. Scoliosis of lower dorsal and lumbar vertebrae with convexity towards the left was observed. The electroencephalogram was normal.

Summarizing the findings, we had a patient with a phenotype that comprised the following: total pancreatic lipomatosis with exocrine insufficiency and endocrine insufficiency (diabetes mellitus), severe bilateral neuropathy (axonopathy) affecting upper and lower limbs (probably a hereditary neuropathy), global developmental delay, intellectual disability, bilateral severe SNHL due to defective OHC functioning, cerebellar atrophy, hypoplastic right vertebral artery, vitamin B12 deficiency, and scoliosis. A syndromic association was strongly suspected and we tried to narrow down the diagnosis.

Pancreatic lipomatosis may occur either secondary to a predisposing cause like ductal calculi or chronic pancreatitis or as a primary condition, in association with certain syndromes. In the former case, it occurs later in life, is focal, and the duct is usually affected. In this patient, the fact that steatorrhoea was evident even from early infancy and that it was present in the elder (deceased) sibling suggests that the pancreatic involvement was likely to be hereditary and congenital. Syndromes associated with pancreatic lipomatosis include cystic fibrosis (CF), Shwachman-Bodian-Diamond Syndrome (SBDS), and Johanson-Blizzard syndrome (JBS). However, these syndromes have additional features such as pulmonary and biliary manifestations in CF, bone marrow insufficiency (especially cyclical neutropenia) and metaphyseal chondrodysplasia in SBDS, and typical facial features and short stature in JBS [7]. These features were not observed in our patient.

Charcot-Marie-Tooth (CMT) disease comprises a group of genetically diverse disorders that share a similar phenotype of hereditary peripheral neuropathy [8]. However, the other features, especially the pancreatic involvement, seen in this patient is not observed in CMT. An association between CMT and co-existing DM has been reported in the literature [9-11]. However, these reports do not include any other feature that was observed in our case.

Having seen that the observed findings in the present case did not fit with any of the above conditions, we proceeded to do a whole exome sequencing (WES). WES revealed the following two variations:

PTRH2 gene - at chromosome 17q23.1 [chr17:57775015] - Exon 3; NM_001015509.2: c.328G>T; p.Glu110* - stop-gain variant - homozygous.

KIF1A gene - at chromosome 2q37.3 [chr2:241727608] - Exon 4; NM_001244008.1: c.223C>T; p.Arg75Trp - missense variant - heterozygous.

The variant identified in the PTRH2 gene is a novel variant, hitherto undescribed, as per the ClinVar and gnomAD (genome aggregation database) databases. In-silico prediction by Mutation Taster, Dynamic Artificial Neural Network (DANN), and Eigen tools predicted the new variant to be ‘deleterious’.

The variant identified in the KIF1A gene is rare as per gnomAD and has been reported to be of unknown significance and pathogenic (by one submitter each) in ClinVar. In-silico tools predicted the variant to be deleterious.

The girl was treated with pancreatic enzyme supplementation and vitamin supplementation. For diabetes, she was continued on insulin. The hypolipidaemia and deficiency of lipid-soluble vitamins were due to pancreatic lipase deficiency. Vitamin B12 deficiency could have been due to pancreatic protease deficiency or it could be an independent feature of the syndrome. The earlier progressive decrease in insulin requirement and episodes of post-prandial hypoglycemia were understood to be due to pancreatic amylase deficiency. It was also due to an eventual improvement in C-peptide levels once the initial glucotoxicity was warded off. Consequent to pancreatic enzyme supplementation, her blood sugar increased transiently due to better absorption of carbohydrates from the diet. Insulin was titrated accordingly. In subsequent visits, it was found that her nutritional status had improved and she is currently on regular follow-up.

## Discussion

In our study, WES revealed a novel, hitherto unreported homozygous, stop-gain type variant in the PTRH2 gene and a heterozygous missense type variant in the KIF1A gene.

The protein encoded by PTRH2 is a highly conserved mitochondrial protein that possesses peptidyl-tRNA hydrolase activity. It helps to release the peptidyl moiety from tRNA, thereby preventing the accumulation of dissociated peptidyl-tRNA, which would otherwise reduce the efficiency of the translation process. The protein also plays a role in cell survival/death regulation. By being a part of the integrin-signaling pathway for cells that attach to the extracellular matrix (ECM), the protein promotes cell survival. However, it also promotes apoptosis in cells that have lost their attachment to the ECM, in a process called anoikis [1].

KIF1A encodes a protein that is a member of the kinesin family and functions as an anterograde transport motor-protein that transports membranous organelles along the microtubules of axons [2].

Variants in PTRH2 were found to be linked to a phenotype of intellectual disability, post-natal microcephaly, progressive ataxia, muscle weakness, demyelinating neuropathy, exocrine pancreas insufficiency, SNHL, and hypothyroidism - in two children of Yazidian-Turkish descent by Hu et al. in 2014 [3]. They named this condition as IMNEPD [Online Mendelian Inheritance in Man (OMIM) #616263]. Subsequently, a few more reports of a similar phenotype associated with variants in PTRH2 were reported [12-16]. These reports presented variable clinical manifestations. Among these, the largest report was that of Picker-Minh et al. who discussed five cases in their paper and identified the ‘core features’ of the IMNEPD syndrome as follows: intellectual disability, motor delay, severe speech delay, ataxia, SNHL, and exocrine pancreatic insufficiency. So far, only 15 patients with IMNEPD phenotype have been described worldwide. The type of the variants - say, missense or nonsense - seems to affect the phenotype. However, variable expressivity with even the same variant among siblings has been observed [15].

Table 1 describes the similar and contrasting features of the phenotype we report in this paper, as compared to that of IMNEPD reported in the literature. The main, as well as contrasting features in our patient, were the prominent, severe axonopathy as compared to the predominant demyelinating neuropathy in IMNEPD, observed OHC dysfunction causing SNHL (cochlear), the clinically severe sensory ataxia that superimposed on cerebellar dysfunction, and the presence of a congenital pancreatic insufficiency that was eventually observed as total pancreatic lipomatosis at 19 years. Additionally, there was hypoplasia of the vertebral artery, scoliosis, and vitamin B12 deficiency. Though the vitamin B12 deficiency could be explained to some extent by pancreatic protease deficiency, it is not evident whether it was a primary manifestation of the disease or a secondary effect of pancreatic insufficiency. Vitamin B12 deficiency was also reported in the paper by Hu et al. (supplementary data). Scoliosis could be a primary defect or a result of muscle weakness.

**Table 1:** A comparison of the clinical phenotype and gene variants present in the syndrome described in this paper (PTRH2 and KIF1A mutations) with the features of previously reported patients with IMNEPD (PTRH2 mutation) N.R.: not reported; +: present; --: not present; (+) ?: doubtful interpretation of feature; ?: not clear from data; N.A.: not applicable; PTRH2: peptidyl t-RNA hydrolase-2 gene; KIF1A: kinesin family member 1A gene: IMNEPD: infantile-onset multisystem, neurologic, endocrine, and pancreatic disease Note: in reports with more than one patient, a feature indicated by the + sign does not mean that it was present uniformly in all patients reported in that paper

Feature	Present study	Hu et al. [3] (2014)	Alazami et al. [12] (2015)	Picker-Minh et al. [13] (2016)	Sharkia et al. [14] (2017)	Le et al. [15] (2019)	Parida et al. [16] (2020)
Genes with observed variants	PTRH2 - homozygous - stop-gain (novel variant) Exon 3; KIF1A - heterozygous - missense Exon 4	PTRH2 - homozygous - deletion and frameshift Exon 2	PTRH2 - homozygous -missense	PTRH2 - homozygous - missense Exon 2	PTRH2 -homozygous -missense	PTRH2 - homozygous - nonsense	PTRH2 -homozygous -single base-pair duplication Exon 1
Number of patients reported	One (with a deceased sibling with a similar phenotype)	Two	One (with a similarly affected sibling, not further described)	Five (including the patient reported by Alazami et al.)	Three	Three	One
Descent	Dravidian-Tamil (Southern India)	Yazidian-Turkish	N.R.	Tunisian and Saudi Arabian	Arabian	Syrian	N.R.
Consanguinity	+	+	+	+	+	+	--
Peripheral neuropathy	+ (Prominent feature in this patient). Severe, predominantly ‘axonal’ sensory and motor neuropathy of upper and lower limbs (hereditary axonopathy)	+ Demyelinating neuropathy	N.R.	+ Demyelinating neuropathy (in one patient. N.R. in others)	+ Demyelinating, axonal polyneuropathy	+ (Nerve conduction showed axonal and demyelinating - in one patient)	+ Demyelinating neuropathy
High-stepping, stomping gait, and sensory ataxia	+ (Prominent feature in this patient)	--	N.R.	? (Ataxia in those patients without cerebellar atrophy is attributed to demyelinating peripheral neuropathy without further objective evidence)	N.R.	+ (Steppage gait in one patient)	N.R.
Romberg’s sign	+	N.R.	N.R.	N.R.	N.R.	N.R.	N.R.
Intellectual disability	+ Moderate intellectual disability	+	+	+ (Core feature of IMNEPD) (Picker-Minh et al.)	--	+	+
Developmental delay and/or regression	+ Global developmental delay	+	+	+ (Core feature of IMNEPD) (Picker-Minh et al.)	+	+	-- No motor delay or isolated language delay
Sensorineural hearing loss	+ Bilateral, severe, sensorineural hearing loss	+	+	+ (Core feature of IMNEPD) (Picker-Minh et al.)	+	+ (Seen in one patient. No hearing loss in two patients)	+
Outer hair cell dysfunction	+ (Prominent feature in this patient) (resulting in bilateral severe, sloping sensorineural hearing loss) - cochlear cause	N.R.	N.R.	N.R.	N.R.	N.R.	N.R.
Pancreatic lipomatosis	+ (Prominent feature in this patient); total pancreatic lipomatosis with preservation of pancreatic duct architecture (probably congenital and progressed)	- Pancreatic fibrosis seen in ultrasound. ? and/or fatty change.	N.R.	+ In one patient. Initial hyperechogenicity on ultrasound (? focal lipomatosis) at 9 years followed by atrophy at 17 years. Not present in others.	--	--	--
Exocrine pancreatic insufficiency	+ (Prominent feature in this patient); severe (secondary to lipomatosis)	+	N.R.	+ (Core feature of IMNEPD) (Picker-Minh et al.)	--	+	--
Diabetes (endocrine pancreatic insufficiency)	+ (Secondary diabetes, secondary to lipomatosis); along with a poor response to hypoglycemia (due to probable glucagon deficiency)	+	N.R.	+	--	+	+
Failure to thrive	+ Due to exocrine pancreatic insufficiency	+	N.R.	+	N.R.	+	--
Hypolipidaemia	+ Due to exocrine pancreatic insufficiency	N.R.	N.R.	N.R.	--	N.R.	--
Deficiency of fat-soluble vitamins	+ Due to exocrine pancreatic insufficiency	+	+ (?) Reported as vomiting blood in the neonatal period (probable vitamin K deficiency)	+	--	N.R.	--
Vitamin B12 deficiency	+ (To some extent it could be due to pancreatic protease deficiency or it could be an independent feature)	+ (Reported in supplemental data)	N.R.	N.R.	--	--	--
Cerebellar atrophy/hypotrophy on imaging	+	+	N.R.	+ (In one patient. Not present in others)	--	+ (Seen in two patients. Not observed in one)	--
Ataxia	+ Not debilitating	+ Progressive and debilitating (making a patient wheelchair-bound)	+	+ (Core feature of IMNEPD) (Picker-Minh et al.). Attributed to demyelinating peripheral neuropathy in the absence of cerebellar atrophy	--	+ Progressive and debilitating (making a patient wheelchair-dependent)	--
Dysmetria	(+) ? Mild dysmetria	+	N.R.	+	--	N.R.	--
Other signs of clinical cerebellar dysfunction	No dysdiadochokinesia. No nystagmus. Tandem walking not possible – due to sensory ataxia and cerebellar cause.	N.R.	N.R.	N.R.	--	N.R.	--
Hypoplastic vertebral artery	+ (Right vertebral artery)	--	N.R.	N.R.	--	--	--
Distal muscle weakness	+ In both upper and lower limbs. Due to neuropathy	+ Debilitating	N.R.	+	+	+ Debilitating	--
Post-natal microcephaly	--	+	?	--	+	+	--
Facial dysmorphism	--	+	--	+ (In one patient. Not present in others)		+	--
Facial palsy	--	--	N.R.	+	--	--	--
Poorly developed teeth	+ (Stained, broken teeth)	N.R.	N.R.	N.R.	N.R.	N.R.	N.R.
Hypotonia	? Mild	+ Neonatal hypotonia	+	+ Neonatal hypotonia (In one patient. Not present in others)	+	+	--
Depressed deep tendon reflexes	+ (Only where peripheral neuropathy was prominent)	N.R.	+	N.R.	+	+	+
Fatty liver	+	+ (Fibrosis or steatosis on ultrasound)	N.R.	+ (In one patient. Not present in others)	--	--	--
Hypothyroidism	--	-- Though reported as ‘hypothyroidism’, the thyroid function tests are within normal limits in the supplementary data provided	N.R.	--	--	--	--
TSH elevation	+ Mild TSH elevation	--	N.R.	+	--	--	--
Seizures	? One episode	--	N.R.	N.R.	--	+	--
EEG abnormality	-- (Normal EEG)	Abnormal rhythmic alpha-beta-waves with high amplitudes on EEG recording	N.R.	N.R.	N.R.	+ (Background slowing; increased asynchronous delta activity - consistent with diffuse encephalopathy; no epileptiform activity)	--
Scoliosis of the vertebral column	+ (Prominent feature in this patient)	--	N.R.	N.R.	--	--	--
Proximal placement of the thumb	--	+	N.R.	+	--	--	--
Long fingers	--	+	N.R.	+	--	--	--
Contracture of tendon Achilles and/or other areas	--	+ (Tendon Achilles)	N.R.	+ (Tendon Achilles)	--	+ (Flexion contractures of hands and feet)	--
Abnormality in the shape of feet	+ Pes cavus, Pes valgus	+ Talipes equinovalgus	+ Club foot	+ Talipes equinovalgus (In one patient. Not present in others)	+ Pes cavus	High foot arches	--
Congenital dislocation of the hip	--	+	--	--	--	--	--
Arachnoid cyst on imaging	--	--	+ Left temporal arachnoid cyst	--	--	--	--
Testicular and scrotal abnormalities	N.A.	+ Shawl scrotum	+ Undescended testis	--	--	+ (Undescended testis in one patient)	N.A.
Delayed menarche	+	N.R.	N.A.	N.R.	+	N.A.	--

PTRH2 knockout mice were observed to be indistinguishable from their healthy littermates at birth but had a runting effect on their growth subsequently. They developed post-natal growth retardation, microcephaly, cerebellar atrophy, and ataxia. Furthermore, the pancreatic acinar area, cortical-neuron soma sizes, hepatocyte, and muscle fiber sizes were found to be reduced. However, the area of the islets of Langerhans was not reduced [3].

Interestingly, even though Hu et al. have mentioned hypothyroidism in the patients they reported, the supplementary data that contains laboratory parameters shows that the thyroid hormone levels including TSH were within normal limits [3]. One could also see from Table 1 that hypothyroidism and/or TSH elevation are not consistent features in the reported cases. Furthermore, hyperglycemia occurs in the IMNEPD patients only secondary to pancreatic dysfunction (secondary diabetes) and not as a primary feature. And, we see that diabetes is also an inconsistent feature in the patients reported. This is in contrast with the predominant involvement of the endocrine system in primary endocrine syndromes like autoimmune polyendocrine syndromes and multiple endocrine neoplasia syndromes. Furthermore, as discussed above, the islet size in the knock-out mice was not reduced before they were sacrificed, while the acinar sizes were reduced [3]. Therefore, the phrase “endocrine and pancreatic” in IMNEPD does not seem to be accurate.

The KIF1A protein is a major molecular-motor protein of the kinesin superfamily involved in axonal transport along the microtubules [17,18]. KRDs include phenotypes that involve the central and peripheral nervous system. The prominent features include spastic paraparesis, axonal sensorimotor neuropathy, congenital contractures, dystonia, dysautonomia, seizures, neurodevelopmental delay, optic atrophy, corpus callosal dysgenesis, and cerebellar atrophy [4,5]. A combination of such findings is sometimes termed as the NESCAV syndrome (OMIM #614255), which stands for neurodegeneration and spasticity with or without cerebellar atrophy or cortical visual impairment. It is caused by heterozygous mutations of KIF1A [19].

Furthermore, KIF1A protein is also found to be localized to the stereociliary bundles and basal bodies of outer and inner hair cells of the organ of Corti in the cochlea [20]. In the patient we described, the predominant axonopathy of upper and lower limb nerves, prominent sensory ataxia, OHC dysfunction, and severe bilateral SNHL, and the history of suspected seizure in childhood may be attributed to the KIF1A gene mutation.

Thus, from the above discussion, we see that the concurrent occurrence of PTRH2 gene and KIF1A gene mutations in our patient (and also probably in her deceased elder brother) made the features of axonal involvement more prominent - like severe peripheral axonopathy, OHC dysfunction, cochlear SNHL, and prominent sensory ataxia. To the best of our knowledge, this is the first report in the medical literature of such a novel syndromic presentation that simultaneously involved both the PTRH2 and KIF1A genes, thus affecting synergistic phenotypical features in the patient.

Proposing the new entity of ‘neuro-pancreatic syndromes’

In clinical medicine, pancreatic disease/insufficiency is known to co-exist - with bone marrow failure (in syndromes like Pearson marrow-pancreas syndrome and SBDS) and - with broncho-pulmonary features in CF. In recent years, a co-existent association between neurological features and pancreatic insufficiency has increasingly been recognized.

Based on the clinical and genetic data reported in the literature so far, and in order to consolidate the findings for practical clinical application, we hereby propose the term - “neuro-pancreatic syndromes” (NPS) as a broad category.

Under NPS, we include the following:

1. The syndrome described by Hu et al. in 2014 (the so-called IMNEPD syndrome) with core features of intellectual disability, demyelinating peripheral neuropathy, cerebellar atrophy, ataxia, SNHL, and exocrine pancreatic insufficiency; it is caused by variation in the PTRH2 gene. As we have mentioned above, endocrine involvement is not a primary or consistent component of the IMNEPD. To make things more precise, we propose an alternative term, the Hu-Matter-Jahns (HMJ) syndrome for IMNEPD.

2. The syndrome described by us in this paper, where a clinical picture of ‘axonal involvement’ - like hereditary axonopathy, OHC dysfunction, cochlear SNHL, and sensory ataxia - predominates along with global developmental delay, intellectual disability, total pancreatic lipomatosis, diabetes, cerebellar atrophy, hypoplasia of vertebral artery and scoliosis - is caused by the synergistic effect of a novel variation in the PTRH2 gene and variation in the KIF1A gene. The predominance of axonal involvement in this scenario may be attributed to the mutation in the KIF1A gene, by which this entity is distinguished from the HMJ syndrome.

As mentioned earlier, the occurrence of diabetes along with a CMT phenotype has been reported [9-11]. But these reports did not have any other specific features of NPS, to include them under NPS. Further studies on phenotypes fitting to the NPS picture may potentially bring out more sub-types under NPS in addition to the two mentioned above.

To have a categorization as NPS is necessary in clinical medicine because of the following reasons: it informs the treating clinician that such a condition exists; once the individual syndromic entities are delineated, the clinical approach becomes easier. Once one or two features are observed, the clinician would be able to anticipate the whole clinical picture and search for the other co-existing conditions. To achieve this, a knowledge of NPS is essential for the treating physician; a prompt and effective diagnosis and management would immensely benefit the patient in terms of treatment and prognosis. Moreover, this would aid researchers including geneticists and clinicians from all over the world to recognize, report, and thereby enhance the knowledge on NPS and also expand the spectrum of NPS.

## Conclusions

In this paper, we described a novel case of combined occurrence of PTRH2 and KIF1A gene variants in a patient, which resulted in a clinical phenotype of global developmental delay, intellectual disability, severe peripheral axonopathy, OHC dysfunction, severe SNHL, total pancreatic lipomatosis, exocrine pancreatic insufficiency, diabetes, cerebellar atrophy, vertebral artery hypoplasia, and scoliosis. To the best of our knowledge, this is the first reported case of such a syndrome due to the synergistic effect of PTRH2 and KIF1A gene mutations in the medical literature.

We also proposed the broader term ‘NPS’, under which we include two entities at present - the syndrome we have presented in this paper, and the IMNEPD syndrome described by Hu et al. We also suggested the term HMJ syndrome for IMNEPD.

We hope that this paper will lead to an enhanced understanding of NPS and trigger more research reports, both clinical and genetic, which would widen the spectrum of NPS in the future, open new doors for treatment options, and ultimately benefit patients with NPS.

## References

[1] (2021). The National Center for Biotechnology Information (NCBI). PTRH2 peptidyl-tRNA hydrolase 2. Gene ID: 51651. https://www.ncbi.nlm.nih.gov/gtr/genes/51651/.

[2] (2021). The National Center for Biotechnology Information (NCBI). KIF1A kinesin family member 1A. Gene ID: 547. https://www.ncbi.nlm.nih.gov/gtr/genes/547/.

[3] Hu H, Matter ML, Issa-Jahns L (2014). Mutations in PTRH2 cause novel infantile-onset multisystem disease with intellectual disability, microcephaly, progressive ataxia, and muscle weakness. Ann Clin Transl Neurol.

[4] Nemani T, Steel D, Kaliakatsos M (2020). KIF1A-related disorders in children: a wide spectrum of central and peripheral nervous system involvement. J Peripher Nerv Syst.

[5] Lee JR, Srour M, Kim D (2015). De novo mutations in the motor domain of KIF1A cause cognitive impairment, spastic paraparesis, axonal neuropathy, and cerebellar atrophy. Hum Mutat.

[6] Bronson SC, Alagianambi S, Mythili C (2021). Syndromic conundrums in diabetes: seek and ye shall find: the Dorfman-Chanarin syndrome. Clin Diabetes.

[7] Weerakkody Y (2021). Radiopaedia. Pancreatic lipomatosis. https://radiopaedia.org/articles/pancreatic-lipomatosis?lang=us.

[8] Amato AA, Barohn RJ (2015). Peripheral neuropathy. Harrison’s Principles of Internal Medicine. 19th Edn.

[9] Ivarsson SA, Bjerre I (1989). Erythrocyte insulin binding in a family with hereditary motor sensory neuropathy (Charcot-Marie-Tooth) with superimposed insulin-dependent type I diabetes mellitus. Diabetes Res.

[10] Yu Z, Wu X, Xie H (2014). Characteristics of demyelinating Charcot-Marie-Tooth disease with concurrent diabetes mellitus. Int J Clin Exp Pathol.

[11] Vega P, Farini ET, Carpio M, Carnelutto N, Chiaradia V, Pisarevsky AA (2017). Diagnosis of Charcot Marie Tooth disease in a patient with type 1 diabetes (Article in Spanish). Medicina (B Aires).

[12] Alazami AM, Patel N, Shamseldin HE (2015). Accelerating novel candidate gene discovery in neurogenetic disorders via whole-exome sequencing of prescreened multiplex consanguineous families. Cell Rep.

[13] Picker-Minh S, Mignot C, Doummar D (2016). Phenotype variability of infantile-onset multisystem neurologic, endocrine, and pancreatic disease IMNEPD. Orphanet J Rare Dis.

[14] Sharkia R, Shalev SA, Zalan A (2017). Homozygous mutation in PTRH2 gene causes progressive sensorineural deafness and peripheral neuropathy. Am J Med Genet A.

[15] Le C, Prasad AN, Rupar CA, Debicki D, Andrade A, Prasad C (2019). Infantile-onset multisystem neurologic, endocrine, and pancreatic disease: case and review. Can J Neurol Sci.

[16] Parida P, Dubbudu A, Biswal SR, Sharawat IK, Panda PK (2021). Diabetes mellitus in an adolescent girl with intellectual disability caused by novel single base pair duplication in the PTRH2 gene: expanding the clinical spectrum of IMNEPD. Brain Dev.

[17] Tanaka Y, Niwa S, Dong M, Farkhondeh A, Wang L, Zhou R, Hirokawa N (2016). The molecular motor KIF1A transports the TrkA neurotrophin receptor and is essential for sensory neuron survival and function. Neuron.

[18] Okada Y, Hirokawa N (1999). A processive single-headed motor: kinesin superfamily protein KIF1A. Science.

[19] Kniffin CL. OMIM #614255 (2021). Online Mendelian inheritance in man (OMIM). #614255 - NESCAV syndrome - NESCAVS. https://www.omim.org/entry/614255.

[20] May-Simera HL, Ross A, Rix S, Forge A, Beales PL, Jagger DJ (2009). Patterns of expression of Bardet-Biedl syndrome proteins in the mammalian cochlea suggest noncentrosomal functions. J Comp Neurol.

